# Supplementary material for: Is resilience linked to stress response among anesthesia professionals? A prospective simulation-based study
Source: PLoS One. 2026 Jun 2;21(6):e0350343. doi: 10.1371/journal.pone.0350343 (PMC13229359; doi:10.1371/journal.pone.0350343)
Supplement: Attachment — Submitted filename: Response_to_Reviewers.docx [file pone.0350343.s002.docx]

**Response to Reviewers**

PONE-D-26-06857

*Is resilience linked to stress response among anesthesia professionals?*

*A prospective simulation-based study.*

Dear Editor and Reviewers,

We sincerely thank the editor and both reviewers for their thorough and constructive evaluation of our manuscript. Their comments have significantly strengthened the paper. Below, we provide a detailed, point-by-point response to each comment. Reviewer comments are presented in *grey italics*, and our responses are in blue text. All line numbers refer to the revised manuscript (clean version).

# Journal Requirements (Staff Editor)

**Requirement 1: PLOS ONE style requirements and file naming**

*Please ensure that your manuscript meets PLOS ONE’s style requirements, including those for file naming.*

We have thoroughly revised the manuscript to comply with PLOS ONE’s formatting and style templates. File naming follows PLOS ONE conventions. All sections are organized according to the required structure (Title, Abstract, Introduction, Methods, Results, Discussion, Acknowledgments, References, Supporting Information).

**Requirement 2: Reference to Figures 2 and 3 in text**

*Please ensure that you refer to Figures 2 and 3 in your text as, if accepted, production will need this reference to link the reader to the figure.*

We have verified that all four figures are explicitly cited in the text. Specifically: Fig 2 is cited in the Results section (line 215: *"Fig 2 illustrates the relationship between resilience and SDNN during the crisis phase"*), and Fig 3 is cited in the Results section (line 225: *"Fig 3 illustrates this relationship"*). These references were already present in the original submission but have been verified for consistency.

**Requirement 3: Anonymization of Supporting Information dataset**

*We note that there is identifying data in the Supporting Information file <S1 dataset resistress.xls>. [...] Please remove or anonymize all personal information (Age), ensure that the data shared are in accordance with participant consent, and re-upload a fully anonymized data set.*

We have fully anonymized the dataset as follows:

(a) Participant IDs containing initials (format "NN-INITIALS") have been replaced with sequential numerical codes (P01 to P34).

(b) Exact ages and years of experience have been removed from the dataset to prevent indirect re-identification, given the small sample size and the specificity of professional groups (e.g., a combination of professional category and years of experience could allow identification in a single-center study).

(c) All hidden columns have been removed. The dataset has been verified to contain no remaining personally identifiable or potentially re-identifying information.

(d) The anonymization approach was reviewed in light of PLOS data policy guidelines and the small sample size. The re-uploaded file is named S1_Dataset.xlsx.

**Requirement 4: Recommended citations**

*If the reviewer comments include a recommendation to cite specific previously published works, please review and evaluate these publications.*

Reviewer #2 recommended the paper by Kagan (2016) "An Overly Permissive Extension" (Perspectives on Psychological Science). We have read and evaluated this paper. It provides a valuable discussion on the imprecise use of psychological constructs, which is directly relevant to our study. We have cited this reference in the revised Introduction (Ref [3]) to support our effort to clearly define the concepts of stress and resilience used in this paper.

# Reviewer #1

**General comment: Turnitin similarity**

*The submitted manuscript is original; however, we suggest reducing the similarity percentage, which according to Turnitin software is 36%, and we note that 0% of the text was generated by AI.*

We thank the reviewer for highlighting the originality of the article. The similarity percentage reported by Turnitin was taken into account. The article has been extensively revised and rewritten, particularly the introduction and discussion sections. A check using the Compilatio software currently shows a similarity percentage of 6%.

**Comment 1: Study objective wording (line 30 of V1)**

*Improve the wording of the study objective, which should be consistent with the study title. The abstract should state a clear and precise objective.*

We have revised the title and the abstract objective for consistency and clarity. The title has been changed from *"How is stress related to resilience in anesthesia professionals?"* to *"Is resilience linked to stress response among anesthesia professionals? A prospective simulation-based study."* The abstract now states a clear hypothesis-driven objective (lines 24–26 of revised manuscript): *"This study aimed to test the hypothesis that higher resilience levels would be associated with attenuated physiological and psychological stress responses during a standardized anesthetic crisis simulation."*

**Comment 2: PSS-10 is not a measure of chronic stress**

*Regarding the instrument used to measure chronic stress, the PSS-10 is not appropriate, as it is a screening test that measures perceived stress. To measure chronic stress in professionals, there are other validated tests such as Maslach’s MBI and others; it is suggested to reformulate this section.*

We fully agree with this important clarification and thank the reviewer for raising it. The PSS-10 measures **perceived stress over the past month**, not chronic stress or burnout. We have corrected this mischaracterization throughout the manuscript. All references to "chronic stress" in relation to the PSS-10 have been replaced with "perceived stress over the past month." Specifically:

• The Introduction now clearly distinguishes between acute stress, perceived stress (PSS-10), and chronic stress/burnout (lines 51–52).

• The Methods section explicitly describes the PSS-10 as measuring "the frequency of situations perceived as threatening" over the past month (line 134).

• The term "chronic stress" is no longer used in association with the PSS-10 anywhere in the manuscript.

• We acknowledge in the Discussion that our study did not include a validated burnout instrument (such as the MBI), and we have removed the previous claim about burnout assessment.

**Comment 3: Methods section – wording, logical coherence, and study design description**

*Improve the wording of the methods section. It is suggested to write in an orderly manner with logical coherence. This is an observational-analytical study that seeks to establish an association. It is necessary to specify the unit of analysis, population, sampling method, sample size, and inclusion criteria. Additionally, the methodology and the validity and reliability of the instruments used should be described. [...] It is also not specified whether the psychological tests were used with permission, were freely available, or were purchased.*

We have substantially reorganized and expanded the Methods section to address all of these concerns. The revised section now follows a logical structure:

1. **Study design**: Clearly described as a prospective, single-center, observational study (line 91-92).

2. **Ethics and registration**: ClinicalTrials.gov registration, ethics approval, and informed consent procedures are specified (lines 92-95).

3. **Population and sampling**: The unit of analysis (individual healthcare professional), sampling method (convenience sampling among volunteers), and the study site are described (lines 99-103). Inclusion/exclusion criteria are detailed (lines 105-108).

4. **Instruments**: Each instrument is described with its psychometric properties from validation studies:

– CD-RISC 10: scoring, range, psychometric properties (α = 0.86), French validation references (line 120)

– PSS-10: scoring convention, French validation (α = 0.83), reverse-scored items (line 139)

– VAS stress: description and references (lines 72)

– Polar H10 / SDNN: accuracy references (lines 122-131)

5. **Authorization**: The revised manuscript states that "Use of the scale was authorized by the original authors" for the CD-RISC 10 (line 121). The PSS-10 is a freely available instrument.

6. **Sample size calculation**: Assumptions and software are specified (line 176-178).

**Comment 4: Data analysis – specify statistical methods**

*In the data analysis section, specify the statistical methods used for descriptive and correlational analysis.*

The Statistical Analysis section has been expanded (lines 178-191). It now specifies: descriptive statistics (medians and quartiles for quantitative variables, counts and percentages for qualitative variables), correlation tests (Pearson or Spearman depending on distribution, with bootstrap confidence intervals for the primary outcome), group comparison tests (Wilcoxon, Student’s t-test, Kruskal–Wallis), and temporal evolution tests (Friedman test). The R version and specific packages used are now listed (line 177).

**Comment 5: Results presentation – table of sample characteristics and restructuring**

*Improve the presentation of results in accordance with the study’s objectives. An initial table of sample characteristics.*

The Results section has been substantially restructured and expanded to align with the study’s objectives and the now hypothesis-driven design. The major changes are as follows:

**a) New Table 2 – Participant characteristics by professional group (lines 198–201):** A descriptive table has been added presenting age, years of experience, prior laryngospasm exposure, CD-RISC 10 scores, PSS-10 scores, basal SDNN, and baseline VAS stress, stratified by the four professional groups (senior anesthesiologists n=9, residents n=9, nurse anesthetists n=10, student nurse anesthetists n=6). Values are reported as median [Q1–Q3].

**b) Internal consistency reported (line 202-203):** Cronbach’s alpha values are now reported for both questionnaires in our sample: CD-RISC 10 α = 0.65 and PSS-10 α = 0.81. The lower-than-expected value for the CD-RISC 10 is discussed in the Limitations section.

**c) Hypothesis-driven structure (lines 212-225):** Results are now organized by hypothesis (H1 through H4), with the primary outcome (H1: CD-RISC 10 vs. SDNN during laryngospasm) clearly identified and reported with bootstrap 95% CI (2,000 resamples). Secondary outcomes (H2–H4) follow in order.

**d) Revised Table 3 – Spearman correlations (lines 232-235):** The correlation table now includes bootstrap 95% CI for all stress measures, the primary outcome is highlighted in bold, and the PSS-10 label has been corrected (previously mislabeled as "chronic stress").

**e) Post-hoc exploratory analyses clearly separated (lines 235-243):** The Kruskal–Wallis comparisons across professional groups and the Friedman tests for temporal evolution of stress markers are now clearly labeled as post-hoc exploratory analyses, distinct from the prespecified hypothesis tests. A caution note is included.

**f) New Fig 4 – Temporal variation (line 244):** A new figure (Fig 4) has been added illustrating the temporal variation of SDNN and VAS stress across simulation phases, confirming that the simulation protocol induced a measurable stress response (Friedman test p < 0.001 for SDNN, p < 0.0001 for VAS).

**Comment 6: Discussion – sampling method and representativeness**

*The discussion states that the sample is representative but does not specify the sampling method; review this aspect and improve the wording.*

We have removed the claim of sample representativeness. The revised manuscript explicitly states that participants were selected by convenience sampling among volunteers (line 102) and acknowledges in the Limitations section (line 289-290) that this convenience sampling limits generalizability. The statement that volunteer bias may affect results is also included.

**Comment 7: Discussion – comparison with theories, recent literature, future research**

*It is suggested to compare the results with theories or explanatory models. [...] It is suggested that the discussion include suggestions for future research, which should consider longitudinal designs and more complex analytical models, such as mediation and moderation analyses.*

The Discussion has been substantially rewritten. It now:

• Frames the null results within the transactional model of stress (Lazarus & Folkman) and the concept of allostatic load (McEwen), explaining why resilience may not attenuate the immediate physiological response (lines 249-258).

• Discusses the well-documented dissociation between physiological biomarkers and self-report measures of stress (lines 253-256).

• Suggests future research directions including: longitudinal designs, larger samples, mediation and moderation analyses between stress, resilience, and performance, and exploration across different cultural contexts and healthcare systems (lines 298-305).

**Comment 8: Dataset completeness**

*The attached dataset is incomplete; only the initial data has been included, and the final data is missing.*

We apologize for this oversight. The revised Supporting Information file (S1_Dataset.xlsx) now includes the complete, fully anonymized dataset with all variables used in the analyses: CD-RISC 10 scores, PSS-10 scores, SDNN measurements at all time points, VAS stress measurements at all time points, and demographic/professional variables (anonymized). Item-level data for questionnaires are included.

# Reviewer #2

*"This is a simulation-training based study aiming to examine the relationship between stress and resiliency in anaesthesia providers. [...] The authors are to be commended for using the simulation lab not only for training but also for research in order to contribute to the stress-performance-personality literature in anesthesia. Overall the paper is concise and reasonably well written."*

We warmly thank Reviewer #2 for this positive assessment and for the insightful suggestions that have substantially improved the manuscript.

**Comment 1: Introduction – Conceptual clarity and definition of terms**

*In the introduction terms such as stress, chronic stress, occupational stress, physiological stress response, psychological trauma and also burnout, resilience and stress management are all used. They are not interchangeable. Psychological concepts such as stress and resiliency need to be clearly defined. I would like to suggest the authors to read this paper: An Overly Permissive Extension. Jerome Kagan. Perspectives on Psychological Science 2016, Vol. 11(4) 442–450.*

We fully agree and have read the recommended paper by Kagan (2016), which we found highly relevant. The Introduction has been entirely rewritten (lines 46-88) to:

• Clearly define stress as encompassing physiological, psychological, and behavioral reactions to a perceived threat, anchored in the transactional model of Lazarus and Folkman [1].

• Explicitly distinguish between acute stress (immediate response), perceived stress (cognitive appraisal), and chronic stress leading to burnout [2,3].

• Define the physiological stress response via the sympathetic nervous system and decreased HRV [4].

• Introduce the concept of allostatic load for chronic exposure (McEwen) [5,6].

• Define resilience as a dynamic process of positive adaptation in the face of adversity [16–18], and explicitly state that resilience does not imply the absence of distress [19].

• Cite Kagan (2016) [3] to support the need for precise definitions and to avoid overly permissive extensions of psychological constructs.

**Comment 2: Introduction – Theoretical framework (transactional model, allostatic load)**

*Furthermore, hypothesized associations between personality characteristics such as resiliency and subsequent stress responses (acute/chronic and physiological/psychological) should be explained within a theoretical framework of stress, eg the transactional stress model (Lazarus and Folkman) or the allostatic load model (McEwan).*

Both the transactional model (Lazarus & Folkman, 1984) and the allostatic load model (McEwen, 2004) are now explicitly integrated into the Introduction. We explain how resilience can be viewed as a personal resource within the transactional model that influences cognitive appraisal (line 69), and how repeated stress exposure can lead to allostatic load with long-term consequences (lines 54-56).

**Comment 3: Introduction – Rewrite entire section, conceptual framework and hypotheses**

*Please rewrite this entire section. Both a conceptual framework and hypothesis are lacking. -What do you mean in this paper when discussing stress? -Why is it important for anesthesiologists and their practice? -Why do you expect an association between physiological stress response, self-reporting stress and self-reported resiliency? What does that mean?*

The Introduction has been entirely rewritten to address all three questions:

1. **What we mean by stress**: We now distinguish acute stress (physiological activation via SNS, decreased SDNN), perceived stress (cognitive appraisal measured by PSS-10), and occupational stress leading to burnout. These are not treated as interchangeable (lines 39–40).

2. **Why it matters for anesthesiologists**: We cite burnout prevalence data (67.7% at high risk, Afonso et al. 2024 [9]) and the impact on cognitive processes and clinical performance (lines 59-60).

3. **Why we hypothesized an association**: Within the transactional model, resilience functions as a personal resource influencing cognitive appraisal. We hypothesized that higher resilience might attenuate physiological and psychological stress responses, while acknowledging that its protective effects might instead operate through longer-term recovery processes (lines 80-82).

4. **Explicit hypotheses**: Four hypotheses are now stated (H1–H4, lines 80-88).

**Comment 4: Methodology – Within-subject design (low-stress vs. high-stress scenarios)**

*Why didn’t you choose to differ the experimental condition within the participant? Ie. confronting each participant with two scenarios: one of which will be a low stress scenario and the other will be a high stress scenario.*

This is an excellent suggestion. A within-subject design with low-stress and high-stress scenarios would indeed strengthen causal inference. However, this was not feasible in our study for several reasons: (1) each simulation session lasts approximately 90 minutes including debriefing, making two sessions per participant logistically challenging; (2) participants were recruited during scheduled training sessions with limited availability; and (3) the educational context of the simulation program did not allow for additional experimental sessions solely for research purposes. We have acknowledged this limitation in the revised Limitations section (lines 296-297): "the study did not compare results across high- and low-stress scenarios, due to logistical constraints and limited participant availability." We agree that future studies should consider within-subject designs.

**Comment 5: Methodology – Psychometric properties of instruments**

*Please provide psychometric properties of questionnaire instruments used.*

Done. The Methods section now reports published psychometric properties for each instrument:

• CD-RISC 10: Cronbach’s α = 0.86, confirmed unidimensional structure (French validations by Guihard et al. [23] and Hébert et al. [24]) – line 66.

• PSS-10: Cronbach’s α = 0.83 (Lesage et al. [29]) – line 70.

In addition, the internal consistency obtained in our own sample is reported in the Results section (line 202-203): CD-RISC 10 α = 0.65, PSS-10 α = 0.81. The lower-than-expected value for the CD-RISC 10 is discussed as a limitation (line 165).

**Comment 6: Methodology – Frequency domain analysis of HRV**

*Did you use frequency domain analysis of HRV? Please elaborate.*

Only time-domain analysis (SDNN) was performed. The revised Methods section now explicitly explains this choice (line 68): "Only time-domain analysis of heart rate variability was performed. Frequency-domain analysis was not conducted, as the 3-minute recording durations per phase were insufficient for reliable spectral analysis in a simulation context involving physical activity." Standard guidelines recommend a minimum of 5 minutes for reliable frequency-domain analysis (Task Force of the ESC and NASPE, 1996), which was not compatible with the duration of individual simulation phases.

**Comment 7: Results – Internal consistency reporting**

*When reporting results of questionnaires often the psychometric properties, internal consistencies, are reported. Why didn’t you do that in your paper?*

We agree this was a significant omission. The Methods section now reports published psychometric properties for each instrument:

• CD-RISC 10: Cronbach’s α = 0.86, confirmed unidimensional structure (French validations by Guihard et al. [23] and Hébert et al. [24]) – line 66.

• PSS-10: Cronbach’s α = 0.83 (Lesage et al. [29]) – line 70.

In addition, the internal consistency obtained in our own sample is reported in the Results section (line 202-203): CD-RISC 10 α = 0.65, PSS-10 α = 0.81. The lower-than-expected value for the CD-RISC 10 is discussed as a limitation (line 165).

**Comment 8: Results – Correction for multiple comparisons and hypothesis formulation (line 165 of V1)**

*Line 165: You report that no correction for multiple comparisons was applied. You explain because it is an exploratory study: why not hypothesize? There is more than enough background if you rewrite your introduction.*

We agree. Following the reviewer’s suggestion, we have formulated four explicit hypotheses (H1–H4) based on the theoretical framework developed in the revised Introduction (lines 80-88). The primary hypothesis (H1: resilience associated with attenuated physiological stress) is now tested with a prespecified analysis including bootstrap confidence intervals. Secondary hypotheses (H2–H4) are clearly labeled. The exploratory post-hoc analyses (Kruskal–Wallis comparisons, Friedman tests) are now clearly distinguished from the hypothesis-driven analyses and are presented separately with a caution note (lines 235-243).

**Comment 9: Results – Isolated association and multiplicity of tests (line 179 of V1)**

*Lines 179: No statistically significant associations were observed between resilience and physiological or perceived stress measures. An isolated association observed in exploratory analyses was not retained after considering the multiplicity of tests. See above: why not hypothesize.*

As described above, the study is now hypothesis-driven. Results are presented by hypothesis (H1 through H4) with the primary outcome clearly identified and tested with bootstrap CI. The isolated association (VAS post-simulation, rho = −0.416, p = 0.015) is discussed with appropriate caution given the absence of correction for multiple comparisons (lines 221-223). Specifically, the revised Discussion now states (lines 267-274): *"A significant isolated association was observed between resilience and perceived stress immediately following the simulation (rho = −0.416, p = 0.015). Although this may suggest that greater resilience is associated with lower perceived stress following an acute event, this finding must be interpreted with great caution. Given the multiple secondary comparisons performed without correction, this association may be a chance finding. Conversely, if confirmed, it could reflect the influence of resilience on the immediate cognitive appraisal of a past stressor. This hypothesis warrants testing in future studies with adequate statistical power and predefined analyses."*

**Comment 10: Discussion – Dissociation between biomarkers and self-report measures**

*Dissociations between biomarker measures of stress and self-reported measures of stress and characteristics of personality are frequently reported in the literature. This paper would be significantly more interesting when discussing why you also found this dissociation.*

This is an excellent point. The revised Discussion now explicitly addresses the dissociation between physiological and self-report measures (line 255): "The relationship between physiological stress markers and self-reported measures is complex and depends on numerous individual factors. SDNN reflects autonomic activation, which exhibits substantial inter-individual variability; this may explain the absence of a direct correlation with resilience." We also discuss that this dissociation is well-documented in the stress literature and does not necessarily invalidate the constructs but rather reflects the multi-dimensional nature of stress.

**Comment 11: Discussion – Relevance of performance (line 216 of V1)**

*Line 216: yes, stress response is important in terms of performance. Is this relevant for your paper? Then please elaborate why here and elsewhere. Performance is not measured in this study. Otherwise keep to the theme.*

We agree. In the revised manuscript, we have substantially reduced mentions of performance to keep the focus on the relationship between stress and resilience. Performance is now mentioned only briefly as a motivation for studying stress in anesthesia professionals in the Introduction (line 59), and as a suggestion for future research in the final paragraph of the Discussion (line 304). The core of the Discussion now focuses on the relationship between acute/perceived stress and resilience, and the dissociation between physiological and psychological measures.

**Comment 12: Discussion – Confusing paragraph, rewrite or omit (line 223 of V1)**

*Line 223 and further. The line of reasoning in this paragraph is confusing. This paragraph seems to be written with an ill understanding of the subject of stress. Concepts are insufficiently defined and connected ad hoc. What do you mean here? This is undermining the paper. Please rewrite or omit.*

We have completely rewritten the Discussion section. The problematic paragraph has been replaced with a coherent argument structured as follows:

1. The null findings are interpreted within the transactional model: resilience modulates cognitive appraisal but does not necessarily attenuate the immediate physiological response, which is largely automatic (lines 249-251).

2. The dissociation between SDNN and self-report measures is discussed as reflecting the multi-dimensional nature of stress (lines 249-251).

3. The observed resilience scores are compared to the literature (lines 259-261).

4. The isolated significant finding (VAS post-simulation) is discussed with appropriate caution (lines 267-270).

5. Implications for screening professionals at psychosocial risk are discussed (Conclusion, lines 310-312).

All concepts are now defined in the Introduction before being used in the Discussion. We believe the revised text no longer suffers from the ad hoc connections that the reviewer identified.

**Comment 13: Conclusion**

*The conclusion should be consistent with the study’s objective. We suggest improving the wording.*

The Conclusion has been rewritten (lines 307-312) to be concise and directly aligned with the study objectives and hypotheses. It now states the main findings (no association between resilience and acute stress measures), the clinical implication (direct measurement of resilience as a complementary tool for identifying professionals at psychosocial risk), and avoids overstatement.

# Summary of Major Changes

**1. Title:** Changed to reflect the hypothesis-driven nature of the study.

**2. Introduction:** Entirely rewritten with: (a) clear definitions of stress types (acute, perceived, chronic/burnout), (b) transactional model of stress (Lazarus & Folkman) and allostatic load (McEwen), (c) Kagan (2016) citation, (d) explicit hypotheses H1–H4.

**3. PSS-10 terminology:** All references to "chronic stress" corrected to "perceived stress over the past month."

**4. Methods:** Restructured with sampling method, population, inclusion/exclusion criteria, psychometric properties of all instruments, authorization/availability of scales, HRV analysis rationale, and detailed statistical methods.

**5. Results:** Added Table 2 (participant characteristics by group), reported Cronbach’s alpha for CD-RISC 10 and PSS-10, structured results by hypothesis, revised Table 3, separated post-hoc analyses, added Fig 4.

**6. Discussion:** Completely rewritten: dissociation between biomarkers and self-report discussed, performance mentions minimized, confusing paragraph removed and replaced, theoretical framework integrated throughout.

**7. Conclusion:** Rewritten for consistency with objectives.

**8. Dataset:** Fully anonymized and complete (S1_Dataset.xlsx).

**9. References:** Added Kagan (2016) [3], McEwen (2004) [5], de Kloet et al. (2005) [6]; updated numbering.

We hope that this comprehensive revision addresses all concerns raised by the editor and reviewers. We remain available for any further clarifications or modifications.

Respectfully,

**Yoann Zafiriou, on behalf of all authors**
